# Financial distress in patients with advanced cancer

**DOI:** 10.1371/journal.pone.0176470

**Published:** 2017-05-18

**Authors:** Cécile Barbaret, Christelle Brosse, Wadih Rhondali, Murielle Ruer, Léa Monsarrat, Patrick Michaud, Anne Marie Schott, Marvin Delgado-Guay, Eduardo Bruera, Stéphane Sanchez, Marilène Filbet

**Affiliations:** 1 Department of Supportive and Palliative Care, Centre Hospitalo-Universitaire de Grenoble, Grenoble, France; 2 Departement of Palliative Care, Institut de Cancérologie de la Loire, Saint-Etienne. France; 3 Department of Supportive and Palliative Care, Centre Hospitalier Lyon-Sud, Lyon. France; 4 Pôle IMER, Hospices Civils de Lyon. France; 5 Department of Palliative Care and Rehabilitation Medicine, MD Anderson Cancer Center, Houston Texas, Unites States of America; 6 Department of Medical Information Evaluation and Performance, Hôpitaux Champagne Sud, Troyes, France; Universita Campus Bio-Medico di Roma, ITALY

## Abstract

**Purpose:**

We examined the frequency and severity of financial distress (FD) and its association with quality of life (QOL) and symptoms among patients with advanced cancer in France.

**Design:**

In this cross-sectional study, 143 patients with advanced cancer were enrolled. QOL was assessed using the Functional Assessment of Cancer General (FACT-G) and symptoms assessed using Edmonton Assessment System (ESAS) and Hospital Anxiety and Depression Scale (HADS). FD was assessed using a self-rated numeric scale from 0 to 10.

**Results:**

Seventy-three (51%) patients reported having FD. Patients reported having FD were most likely to be younger (53.8 (16,7SD) versus 62 (10.5SD), p<0.001), single (33 (62%) versus 40(44%), p = 0.03) and had a breast cancer (26 (36%), p = 0.024). Patients with FD had a lower FACT-G score (59 versus 70, p = 0.005). FD decreased physical (14 versus 18, p = 0.008), emotional (14 versus 16, p = 0.008), social wellbeing (17 versus 19, p = 0.04). Patients with FD had higher HADS-D (8 versus 6 p = 0.007) and HADS-A (9 versus 7, p = 0.009) scores. FD was linked to increased ESAS score (59 (18SD) versus 67 (18SD), p = 0.005) and spiritual suffering (22(29SD) versus 13(23SD), p = 0.045).

**Conclusion:**

The high rate of patient-reported FD was unexpected in our studied population, as the French National Health Insurance covers specific cancer treatments. The FD was associated with a poorer quality of life. Having a systematic assessment, with a simple tool, should lead to future research on interventions that will increase patients’ QOL.

## Introduction

The French healthcare system covers the total cost of specific treatments for cancer patients, but does not include the cost of expenses such as the daily charge for the hospital room, or certain low-risk drugs that are not considered an essential part of cancer treatment. Private insurance provides reimbursement of any such remaining costs incurred by the patient. Persons who cannot afford private insurance due to economic problems are entitled to free ‘top-up’ universal state medical insurance. As specific cancer treatment is free of charge, financial difficulty is considered to be rare in France, and is not systematically assessed. However, there may be other consequences of cancer that affect the family income, such as loss of salary, which can lead to increased financial burden. A study in 2007 [1] in the United States of America found a correlation between the increase of financial difficulties and the decrease of quality of life (QOL) in patients with advanced cancer. In 2016 Perrone et al [2] highlighted the association between financial difficulties with clinical outcomes in a country with public health system. Literature [3,4] highlighted the need to take into account the subjective distress and the consequences of the objective financial burden. Financial distress (FD) is a subjective social class identification that can lead to lower self-esteem and a lower sense of control over life. It has been said that patients withdrawing themselves from healthcare treatment for economic reasons is not uncommon in France [5–7]. Financial difficulties have recently been found to be the most frequent source of distress by cancer patients in a community cancer center in the USA [8]. Despite these results, one of the less explored effects of cancer is its association on finances, the part of FD in the overall suffering and the consequences of FD on QOL. The goal of this study was to evaluate the association of FD on QOL in patients with advanced cancer in France. The primary objective was to determine the association between FD and QOL. Secondary objectives were to characterize the frequency and intensity of FD in advanced cancer patients in France, to evaluate the association between FD and cancer-related symptoms, to determine the association between FD and subjective financial burden, financial worries and concerns and financial difficulties. We hypothesized that FD is associated with poorer QOL and worse psychological and physical symptoms in patients with advanced cancer.

## Method and methods

This cross-sectional survey was approved by the French Institutional Review Board (Centre de Protection des Personnes Lyon Sud-Est; CPP number 12/116, A 13–465; 2012-A01405-38) and registered as a clinical trial NCT01868100. No financial compensation was provided to participate in the study. Written informed consent has been obtained from each patient.

### Definition and assessment

According to literature there are many terms used for describing patients feeling concerning financial issues [9–12]. In this study we used the term FD which defined a subjective experience of distress attributed by the patient to financial burden. Objective financial burden is defined as the percentage of total family income used for direct out-of-pocket expenditure on health care during the last month, which has not been covered by private insurance [13,14] Subjective financial burden is defined as the impact of FD on patients’ wellbeing.

### Population

From the 19^th^ of March 2013 to 30rd of June 2014, 143 patients were enrolled in the Teaching Hospital (TH) Centre Hospitalo-Universitaire de Lyon Sud (CHLS) and a Comprehensive Cancer Centre, Institut de Cancérologie de la Loire (ICL) in Rhône-Alpes. Outpatients or hospitalized patients above 18 years old with advanced lung or breast or colorectal or prostate cancer were included. Advanced cancer was determined by the presence of a relapse, or a metastasis or a second-line-treatment chemotherapy, or a local advanced disease. Non-inclusion criteria were applied to patients presenting with cognitive disorders diagnosed by medical staff. Informed consent was obtained for the patients before the inclusion.

### Data collection and survey instruments

Patient demographics including cancer diagnosis and staging and ECOG (Eastern Cooperative Oncology Group) [15] were collected on chart report. We used the following validated instruments. ESAS (Edmonton Symptom Assessment System) measures the intensity of the most common symptoms within the last 24 hours of cancer patients: pain, fatigue, nausea, depression, anxiety, drowsiness, shortness of breath, appetite, sleep, and wellbeing [16–18]. For this study we added an item assessing spiritual pain. The HADS (Hospital Anxiety and Depression Scale) is a self-assessment tool with 14 items, with two separate subscales for anxiety and depression. A score of 16 or higher is suggestive of a severe case of anxiety or depression [19–21]. FACT-G (Functional Assessment of Cancer Therapy-General) is a 27-question tool validated in cancer patients and has four subscales to assess wellbeing. From these subscales a QOL score is obtained. Both the total score and the individual scores have good internal reliability (alpha = 0.72–0,85)[22,23]. No specific scales exist to assess Financial Burden and FD in France. In this study, financial burden was assessed by questions used in previous studies concerning patients with cancer [24,25]. High burden is defined as more than 10% of total family income spent on out-of-pocket expenses [26,27]. The patients were asked to rate from a “strongly agree to strongly disagree” questions concerning their physical, emotional, social and familial distress. The statement was considered positive when they answered “strongly agree, or agree or somewhat agree”. Subjective financial burden and FD, financial concerns and worries are measured by an exploratory questionnaire. Patients were asked to score a numeric rating scale from 0 to 10 with 0 being best and 10 being worst. Patients were considered having FD if they quoted 1 or above. High FD was defined quoting 4 and above.

### Statistical analysis

For the descriptive analysis, categorical variables were expressed with average and standard deviation (SD), continuous variables with proportion and percentage. The Student T test was used to compare the categorical variables and the chi-square test for the continuous variables. When Student T test or chi-square test could not be used the Mann-Whitney test or Fisher test was applied. One hundred and twenty-five patients were necessary to allow us to detect a Pearson correlation between FD and quality of life as small as 0.25 with 80% power at a 5% type I error rate. A 10% dropout rate was expected so we recruited 143 patients. A cancer-related type stratification was used. We estimated a Pearson and Spearman correlation between FD and quality of life. We compared FD with socioeconomic status using ANOVA or Wilcoxon rank sum tests as appropriate. P values less than 0.05 were considered statistically significant. We used a Bonferroni correction for all statistical analyses. For the statistical analysis SPPS software (Chicago, Inc) 19.0 was used.

## Results

Three hundred and twenty-five patients were screened; of these 124 were not included due to their type of cancer. Twenty-one patients refused to participate. Thirty-seven could not be enrolled due to non-inclusion criteria.

### Baseline characteristics of the patients and patients’ FD (Table 1)

The TH recruited 94 patients (66%) and the CCC 49 patients (34%). Of these, 67 patients (47%) were considered ECOG 2. Seventy (49%) patients were female of whom 42 (29%) reported having FD (p = 0.03). The average age was 58 years old. Patients who reported having FD had a lower average age (53.8 (16,7SD)) than patients with no FD (62 (10.5SD), p<0.001). Fifty-three (37) patients were single. Single patients had more FD (33 (62%)) than those who were married or living with a partner (28 (44%)) (p = 0.003). Eighty-five (59%) of the population did not have a high school leaving qualification. Fifty-three (38%) patients earned less than 15,000 euros a year. Patients earning less than 15,000 euros a year had more FD (34 (64%)) than those earning more (36 (42%), p = 0.013). Patients with breast cancer declared having more FD than patients with other types of cancer (26 (18%) versus 10 (7%), p = 0.024).One hundred and thirty patients (90.9%) had private insurance. Seventy-three (51%) patients declared having FD. Forty-two (30%) had severe FD. Thirty-six (25%) considered their health expenses were greater than they had expected. Thirty-two (22%) said that private insurance did not provide the financial help that they had expected. Twenty-five (17%) patients experienced FD because of healthcare costs. Total family income spent on out-of-pocket expenses went from an average of 6.78 (10.38) before the diagnosis to 9.64 (12.55) after diagnosis. Before diagnosis, 76 (53) patients were employed whereas only 37 (26) were still employed after diagnosis. Patients having FD had a higher average financial burden (p<0.001). Despite these results, there was no significant difference in the percentage of household income spent on health care by patients who reported having FD and those who did not. There was no significant difference between before and after the diagnosis.

**Table 1:** Demographics.

	Financial DistressN = 73 (51%)	No Financial DistressN = 70 (49%)	p	TotalN = 143 (%)
**Patients characteristics**				**N = 143**
Female	42 (29)	28 (20)	0.03	70 (49)
Male	31 (22)	42 (29)		73 (51)
Average age (years) (SD)	53.83(16.7)	62(10.5)	<0.001	
Average time^a^(days) (SD)	1979 (4997)	1619 (1814)	0.57	
**Familial Status**				**N = 143**
*Single*	33 (23)	20 (14)	0.03	53 (37)
*Married*	40 (28)	50 (35)		90 (63)
**Education level**				** N = 143**
*No High school degree*	42 (29)	43 (30)	0.27	85 (59)
*High school degree*	14 (10)	8 (5)		22 (15)
*1–3 y College education*	13 (9)	10 (7)		23 (16)
*>4 y College education*	4 (3)	9 (6)		13 (9)
**Income**				** N = 138**
*< 15000 € a year*	34 (24)	19 (14)	0.013	53 (38)
*> 15000 € a year*	36 (26)	49 (35)		85 (62)
**ECOG**				**N = 143**
*1*	13 (9)	16 (11)	0.16	29 (20)
*2*	35 (25)	32 (22)		67 (47)
*3*	18 (13)	12 (14)		39 (27)
*4*	7 (5)	1 (1)		8 (6)
**Cancer Type**				**N = 143**
*Breast*	26 (18)	10 (7)	0.024	36 (25)
*Lung*	15 (10)	21 (15)		36 (25)
*Prostate*	14 (10)	21 (15)		35 (25)
*Colorectal*	18 (12.5)	18 (12.5)		36 (25)
**Cancer Stage**				**N = 143**
*Metastasis*	64 (45)	58 (40)	0.66	122 (85)
*Relapse*	2 (1)	4 (3)		6 (4)
*Locally Advanced*	5 (3)	7 (5)		12 (8)
*Other*	2 (1)	1 (1)		3 (2)
**Treatments**				**N = 143**
*Surgery*	36 (25)	38 (27)	0.55	74 (52)
*Chemotherapy*	66 (46)	66 (46)	0.38	132 (92)
*Radiotherapy*	38 (27)	42 (29)	0.34	80 (56)
*Targeted Therapy*	5 (4)	3 (2)	0.5	8 (6)

^a^Between the diagnosis and the study.

### Subjective financial burden is shown in Table 2

The average financial concerns and difficulties rates were 3 (SD 3). Patients having FD had more financial difficulty (4.4 (2.94SD), p<0.001), more financial concerns and worries (respectively 4.73 (2.68SD) and 4.55 (2.94SD), p<0.001) that affect their wellbeing (4.61 (2.89SD), p<0.001). Physical (15 (10.6), p<0.001), social (16 (11.3), p = 0.001), emotional (16(11.3), p = 0.001) and spiritual suffering (13 (9.4), p = 0.001) scores were significantly increased in patients having FD. Patients reporting FD had greater anxiety about physical functioning scores than patients without FD (15(10.8), p<0.001).

**Table 2:** Effects of Financial distress on suffering.

	SUBJECTIVE FINANCIAL BURDEN		
	Financial DistressN = 73 (51%)	No Financial DistressN = 70 (49%)	p
Average Financial difficulties (SD)	4.4 (2.94)	0.18 (0.85)	<0.001
Effect on well-being (SD)	4.61 (2.89)	0.25 (1.26)	<0.001
Average Financial Concerns (SD)	4.73 (2.68)	0.92 (2.1)	<0.001
Actual Financial Worries (SD)	4.55 (2.94)	0.42 (1.77)	<0.001
**Effects of financial difficulties on**	**N = 73**	**N = 68**	
Physical suffering	15 (10.6)	1 (1)	<0.001
Anxiety about physical functioning	16 (11.3)	2 (1.4)	0.001
Social suffering	16 (11.3)	2 (1.4)	0.001
Emotional suffering	16 (11.3)	2 (1.4)	0.001
Spiritual suffering	13 (9.4)	1 (1)	0.001

### Association of financial distress on patients’ quality of life and symptoms are shown in Table 3

The average score on the FACT-G was 63 (SD 18). Patients with FD have a lower FACT-G score (59 (18SD) versus 67 (18SD), p = 0.005) especially concerning physical (14 (7SD) versus 18 (7SD) p = 0.008), emotional (14 (6SD) versus 16 (5SD), p = 0.008), and social (17 (5SD) versus 19 (4SD), p = 0.04) wellbeing. Patients with FD had a higher score relating to HADS-D (8(4SD) versus 6 (4SD), p = 0.007) and HADS-A (9 (4SD) versus 7 (4SD), p = 0.009). There were significant differences concerning physical (248 (138SD) versus 177 (132SD), p = 0.02), psychological (106 (73SD) versus 66 (67SD), p = 0.01) and spiritual symptoms (22 (29SD) versus 13 (23SD), p = 0.045) among patients with FD.

**Table 3:** Association of financial distress on patients’ qualities of life.

	Financial DistressN = 73 (51%)	No Financial DistressN = 70 (49%)	p
**QoL Scale**	*Mean (SD)*	*Mean (SD)*	
FACT-G	59 (18)	67 (18)	0.005
PWB^a^	14 (7)	18 (7)	0.008
FSWB^b^	17 (5)	19 (4)	0.04
EWB^c^	14 (6)	16 (5)	0.008
FWB^d^	13 (6)	14 (6)	0.224
HADS-A	9 (4)	7 (4)	0.009
HADS-D	8 (4)	6 (4)	0.007
**ESAS**	*N = 70 Mean (SD)*	*N = 67 Mean (SD)*	
PHS^e^	248 (138)	177 (132)	0.02
PSS^f^	106 (73)	66 (67)	0.01
TSDS^g^	354 (196)	244 (189)	0.01
Spiritual	22 (29)	13 (23)	0.045

^a^PWB: Physical Well Being.

^b^FSWB: Social/Familial Well Being.

^c^EWB: Emotional Well Being.

^d^FWB: Functional Well Being.

^e^PHS: Physical Distress Subscore.

^f^PSS: Psychological Distress Subscore.

^g^TSDS: Total Symptom Distress Subscore.

### Association between patient-reported financial distress and other subjective terms

Table 4 shows the association between the financial distress reported by patients and other subjective terms such as subjective financial burden (r = 0.843, p<0.001), financial concerns (r = 0.791, p<0.001), financial worries (r = 0.816, p<0.001) and financial difficulties (r = 0.88, p<0.001).

**Table 4:** Association between patient reported financial distress and other subjective terms.

	Financial Distress	Subjective Financial Burden	Financial Concerns	Financial Worries	Actual Financial Difficulty
**Financial Distress**					
*Pearson correlation*	1	0.843	0.791	0.816	0.88
*p*		<0.001	<0.001	<0.001	<0.001
**Subjective Financial Burden**					
*Pearson correlation*	0.843	1	0.779	0.825	0.851
*p*	<0.001		<0.001	<0.001	<0.001
**Financial concerns**					
*Pearson correlation*	0.791	0.779	1	0.917	0.78
*p*	<0.001	<0.001		<0.001	<0.001
**Financial worries**					
*Pearson correlation*	0.816	0.825	0.917	1	0.819
*p*	<0.001	<0.001	<0.001		<0.001
**Actual financial difficulty**	0.88	0.851	0.78	0.819	1
*Pearson correlation*	<0.001	<0.001	<0.001	<0.001	
*p*					

## Discussion

This study shows a correlation between FD and QOL and between FD and psychological symptoms (HADS-A and HADS-D). It shows correlation between FD and spiritual suffering and between FD with the ESAS in patients with advanced cancer. Patients with FD had a lower QOL especially concerning physical, emotional, social wellbeing. Those results are confirmed by previous studies [2,28] studying respectively financial distress and financial difficulty and its association with clinical outcomes. Our study is important because it concerns public health system and its impact of reimbursement policy on patients. This study highlighted that financial distress is not protected at least entirely by the French health care system. So further study should focus on other consequences of cancer on patient finances. In an observational study in 2014 [29], Financial Difficulty correlated with social situation, social discrimination and rejection meaning there is a subjective part that we need to assess when studying Financial Difficulty such as FD. Only 21 patients refused to be included which indicates the importance of this subject to French people. Our study highlighted that 73 patients (51%) had FD, which indicates that this is a common problem for the majority of patients with advanced cancer. Patients with FD have higher scores in assessments of anxiety and depression [30]. Depression in patients with advanced cancer is linked to survival rates [31,32]. Our results suggest that the financial situation of patients with advanced cancer should be systemically assessed. Our study highlighted that non-retired patients, breast cancer patients, single persons, women, and patients earning less than 15,000 euros a year were more prone to FD. A previous study confirmed breast cancer patients have more financial difficulties [33]. Further study should therefore be done on FD focusing on this specific population. In France, patients over 62 years of age may be retired with a stable income, which means that financial effect may be less significant. According to our study single patients and women are more prone to have FD. Studies [34,35] highlighted that single persons and single parents are a population more prone to financial burden and poverty. Before the diagnosis, 76 (53) patients had a job whereas after diagnosis only 37 (26) were still employed. Those results are confirmed by a previous study of the French population, which confirms that 8 out of 10 patients had a job before the diagnosis and only 6 were still in employment 2 years later [29]. This study showed that loss of employment was linked to: the younger and older, active population, those with a low level of education, and those engaged in manual work, or work of a precarious nature, and in small businesses. Prognosis influenced work activity. Patients with good prognoses had maintained job activity at 89% (office work) and 74% (physical work), whereas this was 48% and 28% respectively for patients with poor prognoses [29]. We also acknowledge that cancer type influences our results. Except for lung cancer, other types of cancers chosen in our study do not have the poorer prognoses and previous studies showed that poorer prognosis is associated with a higher Financial Difficulty [32–35]. Further study should involve other types of cancers that are more closely linked to difficult socioeconomic status and poorer prognoses. In 2012, the poverty rate in the Rhône-Alpes region was 12.1% (the national rate is 14%) [36,37]. The unemployment rate is above the national one (9.3%). Despite the fact that Rhône-Alpes is one of the richest regions in France our study shows that patients with advanced cancer are prone to have FD. In order to be more representative of the French population, a major study involving all regions of France should be conducted. In recent literature [28], 130 patients out of 147 in the USA had FD. Of 91% patients with insurance, 81% patients and 72% caregivers were experiencing FD. The cost of medical care is a frequent cause of bankruptcy in the USA (46.2% in 2001) and increases every year (62.5% in 2007) [38]. Both the American results and ours highlight that FD is not completely explained by the healthcare system but the French healthcare system seems to offer moderate protection against FD. That may be due to the 100% coverage by French healthcare. This study has some limitations linked to the design of this cross-sectional study. The link between the financial distress and the others factors is not a cause effect relationship. The strong association we observed between FD and the subjective report needs to be confirmed in other studies with larger sample sizes. As only two centers were involved we cannot generalize our results and thus more, larger-scale studies, based on a mix of public and private hospitals, are needed. Furthermore our study used non validated items due to a lack of existing ones so future studies should focus on the validation of items assessing financial distress and financial difficulty. Even if our FD assessment was exploratory, it is an extremely simple way of expressing FD that should trigger a social worker visit. Validation of tools should be made in order to assess FD among patients with cancer and identify interventions to help them. A new tool, Comprehensive Score for Financial Toxicity [39], may be a new lead but needs further investigation. Despite the fact that cancer treatment costs are largely covered by the French healthcare system, this study shows that FD is frequent and is associated with poorer QOL, and more intense psychological, physical and spiritual symptoms. Thus, FD and Financial Difficulty assessment should be systematic.

## Supporting information

S1 Appendix This questiondoinaire was used to interview the patients. (DOCX)

## References

[1] Gupta D, Lis CG, Grutsch JF. Perceived cancer-related financial difficulty: implications for patient satisfaction with quality of life in advanced cancer. Support Care Cancer. 9 2007;15(9):1051–6. doi: 10.1007/s00520-007-0214-2 17242911 10.1007/s00520-007-0214-2

[2] Perrone F, Jommi C, Di Maio M, Gimigliano A, Gridelli C, Pignata S, et al The association of financial difficulties with clinical outcomes in cancer patients: secondary analysis of 16 academic prospective clinical trials conducted in Italy. Ann Oncol. Déc 2016;27(12):2224–9. doi: 10.1093/annonc/mdw433 27789469 10.1093/annonc/mdw433

[3] Selenko E, Batinic B. Beyond debt. A moderator analysis of the relationship between perceived financial strain and mental health. Soc Sci Med. Déc 2011;73(12):1725–32. doi: 10.1016/j.socscimed.2011.09.022 22019305 10.1016/j.socscimed.2011.09.022

[4] Bradshaw M, Ellison CG. Financial hardship and psychological distress: exploring the buffering effects of religion. Soc Sci Med. Juill 2010;71(1):196–204. 20556889 10.1016/j.socscimed.2010.03.015 PMC3770858

[5] ÉTUDE MALADIES GRAVES ET FIN DE VIE DES PERSONN ES EN GRANDE PRECARITE A LYON, GRENOBLE, TOULOUSE ET PARIS [Internet]. [cité 25 oct 2016]. Disponible sur: http://plusdignelavie.com/wp-content/uploads/2012/05/R%C3%A9sum%C3%A9-etude-Pr%C3%A9carit%C3%A9-version-finale.pdf

[6] Négocier ses besoins dans un univers contraint.Le renoncement aux soins en situation de précarité [Internet]. [cité 25 oct 2016]. Disponible sur: http://www.utep-besancon.fr/UTEP_fichup/851.pdf?-session=UTEP_Brb_pref:42F940260edf335BEChqQxE38AC4

[7] Observatoire Régional de la Santé. Rapport final FdF ORS grande precarite_juin11—Grande_precarite.pdf [Internet]. [cité 25 oct 2016]. Disponible sur: http://www.ors-rhone-alpes.org/pdf/Grande_precarite.pdf

[8] Kendall J, Glaze K, Oakland S, Hansen J, Parry C. What do 1281 distress screeners tell us about cancer patients in a community cancer center? Psychooncology. Juin 2011;20(6):594–600. doi: 10.1002/pon.1907 21305646 10.1002/pon.1907

[9] Walson C, Fitzsimmons V. Financial manager’s perception of rural household economic well-being:Development and testing of a composite measure. J Fam Econ Iss. 1993;(14):193–214.

[10] Joo S, Garman E. The relationship between personal financial wellness and employee productivity: A conceptual model. Personal Finances and Worker Productivity. 1998;2:162–71.

[11] Joo S, Grable J. An exploratory framework of the determinnats of financial satisfaction. J Fam Econ Iss. 2004;25:25–50.

[12] Bailey W, Woodiel D, Turner M, Young J. The relationship of financial stress to overall stress and satisfaction. Personal Finances and Worker Productivity. 1998;2:198–207.

[13] Cunningham PJ. The growing financial burden of health care: national and state trends, 2001–2006. Health Aff (Millwood). Mai 2010;29(5):1037–44. 20338908 10.1377/hlthaff.2009.0493

[14] Banthin JS, Cunningham P, Bernard DM. Financial burden of health care, 2001–2004. Health Aff (Millwood). Févr 2008;27(1):188–95. 18180494 10.1377/hlthaff.27.1.188

[15] Oken MM, Creech RH, Tormey DC, Horton J, Davis TE, McFadden ET, et al Toxicity and response criteria of the Eastern Cooperative Oncology Group. Am J Clin Oncol. Déc 1982;5(6):649–55. 7165009

[16] Bruera E, Kuehn N, Miller MJ, Selmser P, Macmillan K. The Edmonton Symptom Assessment System (ESAS): a simple method for the assessment of palliative care patients. J Palliat Care. 1991;7(2):6–9. 1714502

[17] Nekolaichuk C, Watanabe S, Beaumont C. The Edmonton Symptom Assessment System: a 15-year retrospective review of validation studies (1991–2006). Palliat Med. Mars 2008;22(2):111–22. doi: 10.1177/0269216307087659 18372376 10.1177/0269216307087659

[18] Pautex S. Vers une version francophone unique de l’Edmonton Symptom Assessment System (ESAS). 2010 [cité 18 août 2015]; Disponible sur: http://www.chuv.ch/soins-palliatifs/spl_home/spl-recherche/spl-recherches_en_cours.htm

[19] Snaith RP, Zigmond AS. The hospital anxiety and depression scale. Br Med J (Clin Res Ed). 1 Févr 1986;292(6516):344. 10.1136/bmj.292.6516.344 PMC1339318 3080166

[20] Zigmond AS, Snaith RP. The hospital anxiety and depression scale. Acta Psychiatr Scand. Juin 1983;67(6):361–70. 6880820 10.1111/j.1600-0447.1983.tb09716.x

[21] Ravazi D, Delvaux C, Farvacques C, Robaye E. Validation de la version française du HADS dans une population de patients cancéreux hospitalisés. Revue de Psychologie Appliquée. 1989;39(4):295–308.

[22] Costet N, Lapierre V, Benhamou E, Le Galès C. Reliability and validity of the Functional Assessment of Cancer Therapy General (FACT-G) in French cancer patients. Qual Life Res. Juin 2005;14(5):1427–32. 16047518 10.1007/s11136-004-5531-z

[23] Conroy T, Mercier M, Bonneterre J, Luporsi E, Lefebvre JL, Lapeyre M, et al French version of FACT-G: validation and comparison with other cancer-specific instruments. Eur J Cancer. 10 2004;40(15):2243–52. doi: 10.1016/j.ejca.2004.06.010 15454249 10.1016/j.ejca.2004.06.010

[24] Emanuel EJ, Fairclough DL, Slutsman J, Emanuel LL. Understanding economic and other burdens of terminal illness: the experience of patients and their caregivers. Ann Intern Med. 21 Mars 2000;132(6):451–9. 10733444 10.7326/0003-4819-132-6-200003210-00005

[25] Markman M, Luce R. Impact of the cost of cancer treatment: an internet-based survey. J Oncol Pract. Mars 2010;6(2):69–73. doi: 10.1200/JOP.091074 20592778 10.1200/JOP.091074 PMC2835484

[26] Bernard DM, Banthin JS, Encinosa WE. Health care expenditure burdens among adults with diabetes in 2001. Med Care. Mars 2006;44(3):210–5. doi: 10.1097/01.mlr.0000199729.25503.60 16501391 10.1097/01.mlr.0000199729.25503.60

[27] Bernard DSM, Farr SL, Fang Z. National estimates of out-of-pocket health care expenditure burdens among nonelderly adults with cancer: 2001 to 2008. J Clin Oncol. 10 Juill 2011;29(20):2821–6. doi: 10.1200/JCO.2010.33.0522 21632508 10.1200/JCO.2010.33.0522 PMC3139395

[28] Delgado-Guay M, Ferrer J, Rieber AG, Rhondali W, Tayjasanant S, Ochoa J, et al Financial Distress and Its Associations With Physical and Emotional Symptoms and Quality of Life Among Advanced Cancer Patients. Oncologist. 9 2015;20(9):1092–8. doi: 10.1634/theoncologist.2015-0026 26205738 10.1634/theoncologist.2015-0026 PMC4571810

[29] Vie deux ans après diagnostic cancer INSERM [Internet]. [cité 1 août 2015]. Disponible sur: http://www.inserm.fr/content/download/83095/626559/version/1/file/rapport_complet_la-vie-2-ans-apres-un-diagnostic-de-cancer-2014.pdf

[30] Sharp L, Carsin A-E, Timmons A. Associations between cancer-related financial stress and strain and psychological well-being among individuals living with cancer. Psychooncology. Avr 2013;22(4):745–55. doi: 10.1002/pon.3055 22411485 10.1002/pon.3055

[31] Pirl WF, Temel JS, Billings A, Dahlin C, Jackson V, Prigerson HG, et al Depression after diagnosis of advanced non-small cell lung cancer and survival: a pilot study. Psychosomatics. Juin 2008;49(3):218–24. doi: 10.1176/appi.psy.49.3.218 18448776 10.1176/appi.psy.49.3.218

[32] Pirl WF, Greer JA, Traeger L, Jackson V, Lennes IT, Gallagher ER, et al Depression and survival in metastatic non-small-cell lung cancer: effects of early palliative care. J Clin Oncol. 20 Avr 2012;30(12):1310–5. doi: 10.1200/JCO.2011.38.3166 22430269 10.1200/JCO.2011.38.3166 PMC3341144

[33] Arndt V, Merx H, Stürmer T, Stegmaier C, Ziegler H, Brenner H. Age-specific detriments to quality of life among breast cancer patients one year after diagnosis. European Journal of Cancer. 1 Mars 2004;40(5):673–80. doi: 10.1016/j.ejca.2003.12.007 15010067 10.1016/j.ejca.2003.12.007

[34] Les familles monoparentales. Des difficultés à travailler et à se loger [Internet]. [cité 25 oct 2016]. Disponible sur: http://www.insee.fr/fr/ffc/ipweb/ip1195/ip1195.pdf

[35] Les familles monoparentales et leurs conditions de vie—er389.pdf [Internet]. [cité 25 oct 2016]. Disponible sur: http://drees.social-sante.gouv.fr/IMG/pdf/er389.pdf

[36] Insee—Revenus-Salaires—La crise économique creuse les écarts de niveaux de vie en Rhône-Alpes [Internet]. [cité 25 oct 2016]. Disponible sur: http://www.insee.fr/fr/themes/document.asp?reg_id=8&ref_id=20073

[37] Insee—Revenus-Salaires—Rhône-Alpes, une région riche en dépit d’inégalités territoriales [Internet]. [cité 25 oct 2016]. Disponible sur: http://www.insee.fr/fr/themes/document.asp?reg_id=8&ref_id=22969

[38] Himmelstein DU, Thorne D, Warren E, Woolhandler S. Medical bankruptcy in the United States, 2007: results of a national study. Am J Med. Août 2009;122(8):741–6. doi: 10.1016/j.amjmed.2009.04.012 19501347 10.1016/j.amjmed.2009.04.012

[39] de Souza JA, Yap BJ, Hlubocky FJ, Wroblewski K, Ratain MJ, Cella D, et al The development of a financial toxicity patient-reported outcome in cancer: The COST measure. Cancer. 15 10 2014;120(20):3245–53. doi: 10.1002/cncr.28814 24954526 10.1002/cncr.28814

