# Supplementary material for: Financial distress in patients with advanced cancer
Source: PLoS One. 2017 May 18;12(5):e0176470. doi: 10.1371/journal.pone.0176470 (PMC5436643; doi:10.1371/journal.pone.0176470)
Supplement: S1 Appendix — (DOCX) [file pone.0176470.s001.docx]

**Appendix A. Patient Demographics & Baseline Characteristics**

**Gender**: ❒ Female ❒ Male

**Date of Birth**: ____________________

**Ethnicity:**

⃞ White ⃞ Asian/Pacific Islander ⃞ Black (African American) ⃞ Hispanic

⃞ American Indian/Native American ⃞ Other____________

**Marital status:**

⃞ Single ⃞ Married ⃞ Widowed ⃞ Divorced ⃞ Separated ⃞ Other: _________

**Religion**:

Catholic  Christian/Protestant  Jewish  Muslim  Other____________

**Education:**

 High school or less  Basic college education  Advanced degree

**Cancer Diagnosis:**

Date of diagnosis: ____________________

Type: ⃞ Breast ⃞ Lung ⃞ Prostate ⃞ Colorectal

Stage: ⃞ Metastatic ⃞ Recurrent ⃞ Locally-advanced ⃞ Other: ________

**Current treatment**:

⃞ Chemotherapy ⃞ Radiation ⃞ Targeted therapy or Phase 1

**ECOG Performance Status**: ⃞ 1 ⃞ 2 ⃞ 3 ⃞ 4

**Appendix B. The Socioeconomic Status Instrument**

The following is a short questionnaire that would help with the proposed study. Please answer the questions as completely as possible. We appreciate your time in filling out these forms and welcome any comments or questions. As always, we strive to protect your privacy and all records are confidential.

| 1. **How many children less than 18 years of age live in your household?** | | Codes |
| --- | --- | --- |
|  | Please, state the number of children _____ |  |
|  | None | 0 |
|  | Refused to answer | -9 |
|  |  |  |
| **2. What is the highest grade or year of school you completed?** | |  |
|  | Never attended school or only attended kindergarten | 1 |
|  | Grades 1 through 8 (Elementary) | 2 |
|  | Grades 9 through 11 (some High school) | 3 |
|  | Grades 12 or GED (High school graduate) | 4 |
|  | College 1 year to 3 years (some College or technical school) | 5 |
|  | College 4 years or more (College graduate) | 6 |
|  |  |  |
| **3. Are you currently...?** | |  |
|  | Employed for wages | 1 |
|  | Self-employed | 2 |
|  | Out of work for more than 1 year | 3 |
|  | Out of work for less than 1 year | 4 |
|  | A homemaker | 5 |
|  | A student | 6 |
|  | Retired | 7 |
|  | Unable to work | 8 |
|  | Refused to answer | -9 |
|  |  |  |
| **4. Is your annual household income from all sources before taxes…?** | |  |
|  | Less than 10,000 | 1 |
|  | $10,000 to less than $15,000 | 2 |
|  | $15,000 to less than $20,000 | 3 |
|  | $20,000 to less than $25, 000 | 4 |
|  | $25,000 to less than $35,000 | 5 |
|  | $35,000 to less than $50,000 | 6 |
|  | $50,000 to less than $75,000 | 7 |
|  | $75,000 or more | 8 |
|  |  |  |
| **5. Do you have more than one telephone number in your household? (Do not include cell phones or numbers that are only used by a computer or fax machine.)** | |  |
|  | 1. Yes | 1 |
|  | 1. No | 2 |
|  | 1. Don’t know/Not sure | -8 |
|  | 1. Refused | -9 |

| **6. How many of these telephone numbers are residential numbers** | | Codes |
| --- | --- | --- |
|  | Please, state the number of residential telephones: ________ |  |
|  | Don’t know/Not sure | -8 |
|  | Refused | -9 |
|  |  |  |
| **7. During the past 12 months, has your household been without telephone service for 1 week or more? (Do not include interruptions of telephone service because of weather or natural disasters.)** | |  |
|  | Yes | 1 |
|  | No | 2 |
|  | Don’t know/Not sure | -8 |
|  | Refused | -9 |
|  |  |  |
| **8. Was there a time in the past 12 months when you needed to see a doctor but could not because of the cost?** | |  |
|  | Yes | 1 |
|  | No | 2 |
|  | Don’t know/Not sure | -8 |
|  | Refused | -9 |
|  |  |  |
| **9. Which county do you live in?** | |  |
|  | Please, state the name of your county: _________________________________ and State: __________ |  |
|  | Don’t know/Not sure | -8 |
|  | Refuse | -9 |
| **FOR THE RESEARCH PERSONNEL ONLY** | | |
| Using the responses to question number 9 and the links provided below, kindly derive the following data. | | |
| **10. Urbanization code of the patient’s County of Residence:** | | |
|  | 1 | |
|  | 2 | |
|  | 3 | |
|  | 4 | |
|  | 5 | |
|  | 6 | |
|  | 7 | |
|  | 8 | |
|  | 9 | |
|  | 10 | |
|  | 11 | |
|  | 12 | |
|  |  | |
| **11. Median household income of the patient’s County of residence is** $__________ | | |
|  |  | |
| **12. Percentage of persons living below the poverty level in the patient’s County of residence is** ______________% | | |

***Please, see below.***

**Helpful information on how to use this instrument**

Links:

- For Urbanization code, kindly see: 2003 Urban Influence Codes [Internet]. Washington (DC): Economic Research Services. The United States Department of Agriculture. Available from: <http://ers.usda.gov/Data/UrbanInfluenceCodes/2003/>
- For County statistics, kindly see: State and County Quickfacts. [Internet]. Washington (DC). U.S. Bureau of Census. Available from: <http://quickfacts.census.gov/qfd/index.html>

**Appendix C: FINANCIAL BURDEN**

Please estimate **the financial burden,** ***since your cancer diagnosis.***

1. **Without giving exact dollars, how would you describe your household’s financial situation right now?  Would you say that :**

❒ You are having difficulty paying the bills not matter what you do.

❒ You have money to pay the bills, but only because you have cut back

on things.

❒ You have enough money o pay the bills but little spare money to buy

extra or special things.

❒ After paying the bills, you still have enough money for special things

that you want.

1. **How would you describe your reaction to the cost of treating cancer?**

❒ Spent more money than I expected

❒ Spent about what I expected

❒ Spent less than I expected

1. **How would you describe your reaction to how your medical benefits covered the cost of treating cancer?**

❒ Contributed more money than I expected

❒ Contributed about what I expected

❒ Contributed less money than I expected

1. **Has the financial cost of treating your cancer caused you or your family distress?**

❒ No ❒ Yes, a small amount ❒ Yes, a large amount

1. **When choosing a treatment course for your cancer, did you consider the cost of treatment?**

❒ No, not at all ❒ Yes, a little bit ❒ Yes, a great deal

1. **Did you decide not to have a recommended cancer treatment because it was too expensive?**

❒ No ❒ Yes ❒ Do not know/not sure

1. **Please rate the financial burden that you and your family have experienced related to the cost of your illness and medical care**

No Financial Burden Worst Financial Burden

_________________________________________________________

0 1 2 3 4 5 6 7 8 9 10

**BEFORE YOU WERE DIAGNOSED WITH CANCER:**

1. **Your insurance status was:**

❒ Private Insurance: □ HMO □ PPO

❒ Fee for Service (FFS)

❒ Medicare only

❒ Medicare plus supplement

❒ Medicaid

❒ VA or Military

❒ Uninsured or indigent

❒ Other: ____________________

1. **Were you employed or had a job?**

❒ No

❒ Yes

1. **On average, what was your estimated monthly household income? (consider an average of three months)**

❒ < 2500 USD

❒ Between 2500 and 5000 USD

❒ Between 5000 and 7500 USD

❒ Between 7500 and 10000 USD

❒ > 10000 USD

1. **On average, before you were diagnosed with cancer, what percentage of your family total monthly income was spent in health related issues (including out-of pocket payments for prescriptions and office visits)?**

**______________________________________________________________________**

**0% 5 10 15 20 25 30 35 40 45 50 55 65 70 75 80 85 90 95 100%**

1. **Which class do you assign yourself to?**

**Working Class (WC)**

❒ **Lower (LWC)**

❒  **Middle (MWC)**

❒ **Upper (UWC)**

**Upper class (UC):**

❒ **Lower (LUC)**

❒ **Middle (MUC)**

❒ **Upper (UUC)**

**Middle Class (MC)**

❒ **Lower (LMC)**

❒ **Middle (MMC)**

❒ **Upper (UMC)**

**THE FOLLOWING QUESTIONS ARE RELATED TO YOUR SITUATION TODAY**

1. **Your insurance status is :**

❒ Private Insurance: □ HMO □ PPO

❒ Fee for Service (FFS)

❒ Medicare only

❒ Medicare plus supplement

❒ Medicaid

❒ VA or Military

❒ Uninsured or indigent

❒ Other: ____________________

1. **Are you employed or have a job?**

❒ No

❒ Yes

1. **What is your estimated monthly household income? (Consider an average for the last 3 months).**

❒ < 2500 USD

❒ Between 2500 and 5000 USD

❒ Between 5000 and 7500 USD

❒ Between 7500 and 10000 USD

❒ > 10000 USD

1. **On average, what percentage of your family total monthly income was spent in health related issues (including out-of pocket payments for prescriptions and office visits) during the last three months?**

**______________________________________________________________________**

**0% 5 10 15 20 25 30 35 40 45 50 55 65 70 75 80 85 90 95 100%**

1. **Which class do you assign yourself to?**

**Working Class (WC)**

❒ **Lower (LWC)**

❒  **Middle (MWC)**

❒ **Upper (UWC)**

**Upper class (UC):**

❒ **Lower (LUC)**

❒ **Middle (MUC)**

❒ **Upper (UUC)**

**Middle Class (MC)**

❒ **Lower (LMC)**

❒ **Middle (MMC)**

❒ **Upper (UMC)**

**Appendix D: FINANCIAL DISTRESS**

- 1. Please circle the number that best describes your level of financial distress within the past month.

No Financial Distress Worst Financial Distress

_______________________________________________________

0 1 2 3 4 5 6 7 8 9 10

- 1. How much do you feel your financial stress is affecting your well being over the past month?

Not all very much

_________________________________________________________

0 1 2 3 4 5 6 7 8 9 10

**Please give us your opinion on the following statements about your current financial distress.**

- 1. I have more financial distress than physical distress (pain, fatigue, nausea, etc).

Strongly Somewhat Somewhat Strongly

disagree disagree Neutral agree agree

❒ ❒ ❒ ❒ ❒

- 1. I have more financial distress than distress about my physical functioning.

Strongly Somewhat Somewhat Strongly

disagree disagree Neutral agree agree

❒ ❒ ❒ ❒ ❒

- 1. I have more financial distress than social/family distress.

Strongly Somewhat Somewhat Strongly

disagree disagree Neutral agree agree

❒ ❒ ❒ ❒ ❒

- 1. I have more financial distress than emotional distress (depression, anxiety).

Strongly Somewhat Somewhat Strongly

disagree disagree Neutral agree agree

❒ ❒ ❒ ❒ ❒

- 1. I have more financial distress than spiritual distress.

Strongly Somewhat Somewhat Strongly

disagree disagree Neutral agree agree

❒ ❒ ❒ ❒ ❒

- 1. Financial difficulties

No Financial difficulties Worst Financial difficulties

_________________________________________________________

1. 1 2 3 4 5 6 7 8 9 10
   1. Financial concerns

No Financial concerns Worst Financial concerns

_________________________________________________________

1. 1 2 3 4 5 6 7 8 9 10
   1. Financial worries

No Financial worries Worst Financial worries

_________________________________________________________

0 1 2 3 4 5 6 7 8 9 10

**Appendix E: EDMONTON SYMPTOM ASSESSMENT SCALE (ESAS)**

**Appendix F: THE HOSPITAL ANXIETY AND DEPRESSION SCALE**


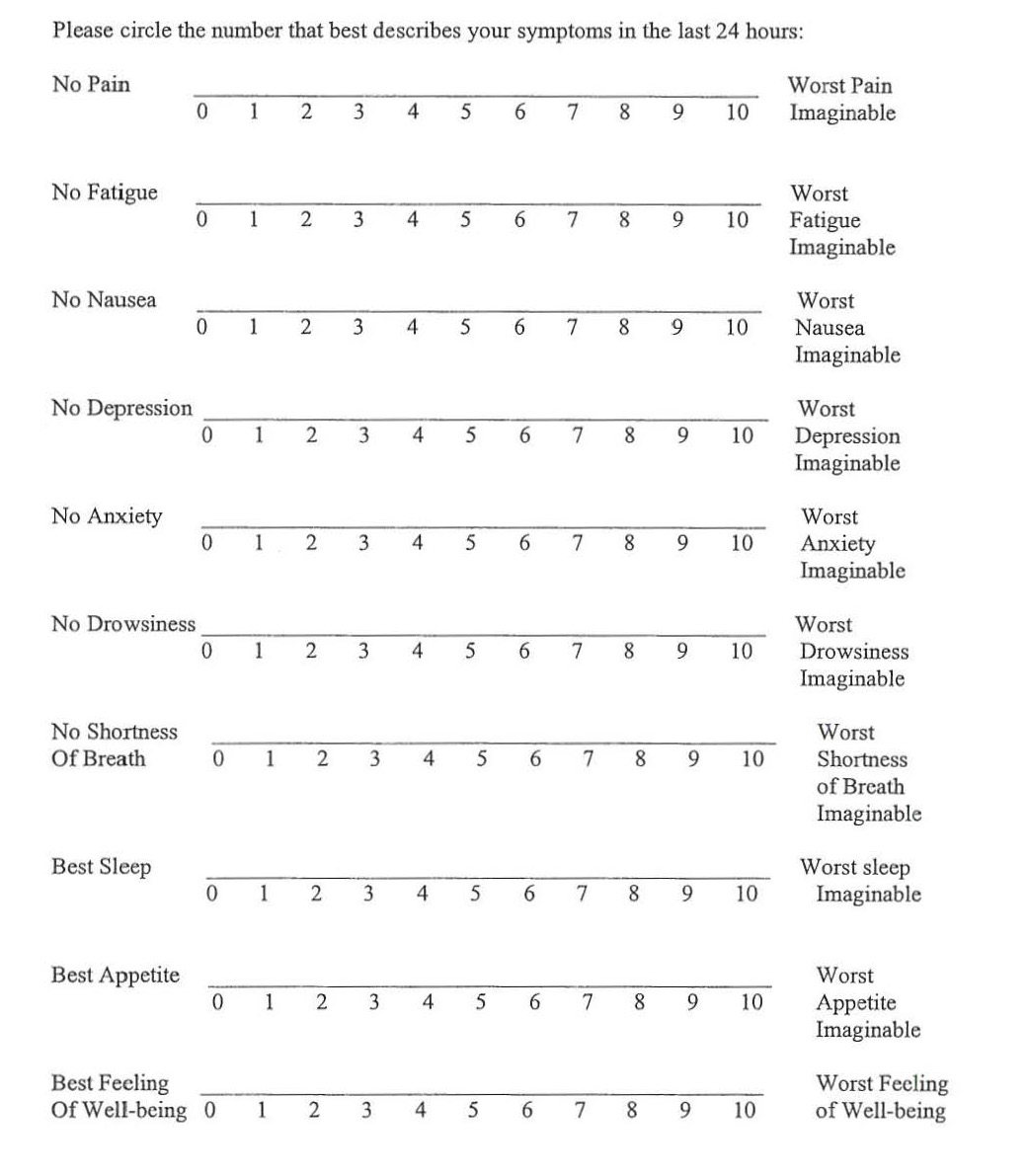


(Please underline the reply which comes closest to how you have been feeling in the past week).

1. **I feel tense or ‘wound up’:**

Most of the time

A lot of the time

From time to time, occasionally

Not at all

2. **I still enjoy the things I used to enjoy:**

Definitely as much

Not quite so much

Only a little

Hardly at all

3. **I get sort of frightened feeling as if something awful is about to happen**:

Very definitely and quite badly

Yes, but not too badly

A little, but it doesn’t worry me

Not at all

4. **I can laugh and see the funny side of things:**

As much as I always could

Not quite so much now

Definitely not so much now

Not at all

5. **Worrying thoughts to go through my mind:**

A great deal of the time

A lot of the time

From time to time but not too often

Only occasionally

6. **I feel cheerful**:

Not at all

Not often

Sometimes

Most of the time

7. **I can sit at ease and feel relaxed**:

Definitely

Usually

Not often

Not at all

8. **I feel as if I am slowed down**:

Nearly all the time

Very often

Sometimes

Not at all

9. **I get a sort of frightened feeling like ‘butterflies’ in the stomach:**

Not at all

Occasionally

Quite often

Very often

10. **I have lost interest in my appearance:**

Definitely

I don’t take so much care as I should

I may not take quite as much care

I take just as much care as ever

11. **I feel restless as if I have to be on the move:**

Very much indeed

Quite a lot

Not very much

Not at all

12. **I look forward with enjoyment to things**:

As much as ever I did

Rather less than I used to

Definitely less than I used to

Hardly at all

13. **I get sudden feelings of panic**:

Very often indeed

Quite often

Not very often

Not at all

14. **I can enjoy a good book or radio or TV program:**

Often

Sometimes

Not often

Very seldom

**Appendix G: SPIRITUAL DISTRESS ASSESSMENT TOOL**

**Meaning in Life Questionnaire**

**Instruction:** Read each item carefully and “x” mark the column that best indicates your values and beliefs.

|  | Disagree strongly  1 | Disagree  2 | Agree  3 | Agree strongly  4 |
| --- | --- | --- | --- | --- |
| 1. **I have a system of values and beliefs that guide my daily activities.** |  |  |  |  |
| 1. **I have a philosophy of life that helps me understand who I am.** |  |  |  |  |
| 1. **I feel like I am living fully.** |  |  |  |  |
| 1. **I feel I have found a really significant meaning in my life.** |  |  |  |  |
| 1. **In my life, I have clear goals and aims.** |  |  |  |  |
| 1. **I have a sense of direction and purpose in life.** |  |  |  |  |
| 1. **I feel good when I think of what I have done in the past.** |  |  |  |  |
| 1. **I am at peace with my past.** |  |  |  |  |

❒ ❒ ❒ ❒

**Appendix H: FACIT-sp-pal**

Below is a list of statements that other people with your illness have said are important**. Please circle or mark one number per line to indicate your response as it applies to the past 7 days.**

|  | **PHYSICAL WELL-BEING** | **Not at all** | **A little bit** | **Some-what** | **Quitea bit** | **Very much** |
| --- | --- | --- | --- | --- | --- | --- |
|  |  |  |  |  |  |  |
| GP1 | I have a lack of energy | 0 | 1 | 2 | 3 | 4 |
| GP2 | I have nausea | 0 | 1 | 2 | 3 | 4 |
| GP3 | Because of my physical condition, I have trouble meeting the needs of my family | 0 | 1 | 2 | 3 | 4 |
| GP4 | I have pain | 0 | 1 | 2 | 3 | 4 |
| GP5 | I am bothered by side effects of treatment | 0 | 1 | 2 | 3 | 4 |
| GP6 | I feel ill | 0 | 1 | 2 | 3 | 4 |
| GP7 | I am forced to spend time in bed | 0 | 1 | 2 | 3 | 4 |
|  | **SOCIAL/FAMILY WELL-BEING** | **Not at all** | **A little bit** | **Some-what** | **Quitea bit** | **Very much** |
|  |  |  |  |  |  |  |
| GS1 | I feel close to my friends | 0 | 1 | 2 | 3 | 4 |
| GS2 | I get emotional support from my family | 0 | 1 | 2 | 3 | 4 |
| GS3 | I get support from my friends | 0 | 1 | 2 | 3 | 4 |
| GS4 | My family has accepted my illness | 0 | 1 | 2 | 3 | 4 |
| GS5 | I am satisfied with family communication about my illness | 0 | 1 | 2 | 3 | 4 |
| GS6 | I feel close to my partner (or the person who is my main support) | 0 | 1 | 2 | 3 | 4 |
| Q1 | *Regardless of your current level of sexual activity, please answer the following question. If you prefer not to answer it, please mark this box and go to the next section.* |  |  |  |  |  |
| GS7 | I am satisfied with my sex life | 0 | 1 | 2 | 3 | 4 |

|  | **EMOTIONAL WELL-BEING** | **Not at all** | **A little bit** | **Some-what** | **Quitea bit** | **Very much** |
| --- | --- | --- | --- | --- | --- | --- |
|  |  |  |  |  |  |  |
| GE1 | I feel sad | 0 | 1 | 2 | 3 | 4 |
| GE2 | I am satisfied with how I am coping with my illness | 0 | 1 | 2 | 3 | 4 |
| GE3 | I am losing hope in the fight against my illness | 0 | 1 | 2 | 3 | 4 |
| GE4 | I feel nervous | 0 | 1 | 2 | 3 | 4 |
| GE5 | I worry about dying | 0 | 1 | 2 | 3 | 4 |
| GE6 | I worry that my condition will get worse | 0 | 1 | 2 | 3 | 4 |

|  | **FUNCTIONAL WELL-BEING** | **Not at all** | **A little bit** | **Some-what** | **Quitea bit** | **Very much** |
| --- | --- | --- | --- | --- | --- | --- |
|  |  |  |  |  |  |  |
| GF1 | I am able to work (include work at home) | 0 | 1 | 2 | 3 | 4 |
| GF2 | My work (include work at home) is fulfilling | 0 | 1 | 2 | 3 | 4 |
| GF3 | I am able to enjoy life | 0 | 1 | 2 | 3 | 4 |
| GF4 | I have accepted my illness | 0 | 1 | 2 | 3 | 4 |
| GF5 | I am sleeping well | 0 | 1 | 2 | 3 | 4 |
| GF6 | I am enjoying the things I usually do for fun | 0 | 1 | 2 | 3 | 4 |
| GF7 | I am content with the quality of my life right now | 0 | 1 | 2 | 3 | 4 |

|  | **ADDITIONAL CONCERNS** | **Not at all** | **A little bit** | **Some-what** | **Quite**  **a bit** | **Very much** |
| --- | --- | --- | --- | --- | --- | --- |
|  |  |  |  |  |  |  |
| Sp1 | I feel peaceful | 0 | 1 | 2 | 3 | 4 |
| Sp2 | I have a reason for living | 0 | 1 | 2 | 3 | 4 |
| Sp3 | My life has been productive | 0 | 1 | 2 | 3 | 4 |
| Sp4 | I have trouble feeling peace of mind | 0 | 1 | 2 | 3 | 4 |
| Sp5 | I feel a sense of purpose in my life | 0 | 1 | 2 | 3 | 4 |
| Sp6 | I am able to reach down deep into myself for comfort | 0 | 1 | 2 | 3 | 4 |
| Sp7 | I feel a sense of harmony within myself | 0 | 1 | 2 | 3 | 4 |
| Sp8 | My life lacks meaning and purpose | 0 | 1 | 2 | 3 | 4 |
| Sp9 | I find comfort in my faith or spiritual beliefs | 0 | 1 | 2 | 3 | 4 |
| Sp10 | I find strength in my faith or spiritual beliefs | 0 | 1 | 2 | 3 | 4 |
| Sp11 | My illness has strengthened my faith or spiritual beliefs | 0 | 1 | 2 | 3 | 4 |
| Sp12 | I know that whatever happens with my illness, things will be okay | 0 | 1 | 2 | 3 | 4 |
| PAL1 | I maintain contact with my friends | 0 | 1 | 2 | 3 | 4 |
| PAL2 | I have family members who will take on my responsibilities | 0 | 1 | 2 | 3 | 4 |
| PAL3 | I feel that my family appreciates me | 0 | 1 | 2 | 3 | 4 |
| PAL4 | I feel like a burden to my family | 0 | 1 | 2 | 3 | 4 |
| B1 | I have been short of breath | 0 | 1 | 2 | 3 | 4 |
|  |  | **Not at all** | **A little bit** | **Some-what** | **Quite**  **a bit** | **Very much** |
| PAL5 | I am constipated | 0 | 1 | 2 | 3 | 4 |
| C2 | I am losing weight | 0 | 1 | 2 | 3 | 4 |
| O2 | I have been vomiting | 0 | 1 | 2 | 3 | 4 |
| PAL6 | I have swelling in parts of my body | 0 | 1 | 2 | 3 | 4 |
| PAL7 | My mouth and throat are dry | 0 | 1 | 2 | 3 | 4 |
| Br7 | I feel independent | 0 | 1 | 2 | 3 | 4 |
| PAL8 | I feel useful | 0 | 1 | 2 | 3 | 4 |
| PAL9 | I make each day count | 0 | 1 | 2 | 3 | 4 |
| PAL10 | I have peace of mind | 0 | 1 | 2 | 3 | 4 |
| Sp21 | I feel hopeful | 0 | 1 | 2 | 3 | 4 |
| PAL12 | I am able to make decisions | 0 | 1 | 2 | 3 | 4 |
| L1 | My thinking is clear | 0 | 1 | 2 | 3 | 4 |
| PAL13 | I have been able to reconcile (make peace) with other people | 0 | 1 | 2 | 3 | 4 |
| PAL14 | I am able to openly discuss my concerns with the people closest to me | 0 | 1 | 2 | 3 | 4 |
